# Association between Perfluorinated Compound Exposure and Miscarriage in Danish Pregnant Women

**DOI:** 10.1371/journal.pone.0123496

**Published:** 2015-04-07

**Authors:** Tina Kold Jensen, Louise Bjørkholt Andersen, Henriette Boye Kyhl, Flemming Nielsen, Henrik Thybo Christesen, Philippe Grandjean

**Affiliations:** 1 Department of Environmental Medicine, University of Southern Denmark, Odense, Denmark; 2 Odense Child Cohort, Hans Christian Andersen Children’s Hospital, Odense, Denmark; 3 OPEN (Odense Patient data Explorative Network), Odense University Hospital, Odense, Denmark; 4 Hans Christian Andersen Children’s Hospital, Odense University Hospital, Odense, Denmark; 5 Institute of Clinical Research, University of Southern Denmark, Odense, Denmark; Gentofte University Hospital, DENMARK

## Abstract

Perfluorinated alkylated substances (PFAS) have been extensively used in consumer products and humans are widely exposed to these persistent compounds. A recent study found no association between exposure to perfluorooctanoic acid (PFOA) and perfluorooctanesulfonic acid (PFOS) and miscarriage, but no studies have examined adverse effect of the more recently introduced PFASs. We therefore conducted a case-control study within a population-based, prospective cohort during 2010-2012. Newly pregnant women residing in the Municipality of Odense, Denmark were invited to enroll in the Odense Child Cohort at their first antenatal visit before pregnancy week 12. Among a total of 2,874 participating women, 88 suffered a miscarriage and 59 had stored serum samples, of which 56 occurred before gestational week 12. They were compared to a random sample (N=336) of delivering women, who had also donated serum samples before week 12. Using a case-control design, 51 of the women suffering a miscarriage were matched on parity and gestational day of serum sampling with 204 delivering women. In a multiple logistic regression with adjustment for age, BMI, parity and gestational age at serum sampling, women with the highest tertile of exposure to perfluorononanoic acid (PFNA) and perfluorodecanoic acid (PFDA) in pregnancy had odds ratios for miscarriage of 16.5 (95% CI 7.4-36.6-36.5) and 2.67 (1.31-5.44), respectively, as compared to the lowest tertile. In the matched data set, the OR were 37.9 (9.9-145.2) and 3.71 (1.60-8.60), respectively. The association with perfluorohexane sulfonic acid (PFHxS) was in the same direction, but not statistically significant, while no association was found with PFOA and PFOS. Our findings require confirmation due to the possible public health importance, given that all pregnant women are exposed to these widely used compounds.

## Introduction

Perfluorooctanoic acid (PFOA) and perfluorooctane sulfonate (PFOS) are perfluorinated alkylated substances (PFAS), which are widely used in consumer products and industrial applications because of their stain-, grease- and water-resistant properties. PFOA and PFOS have relatively long elimination half-lives in humans (3.8–5.4 years). Their use is being phased out in favor of PFAS with carbon chains either shorter or longer than eight.[1] Dietary exposure, particularly through seafood (PFOS) and leaching from food packaging materials, may be a major exposure route, and nearly all serum samples from the general population contain detectable concentrations of both PFOA and PFOS.[2, 3] PFAS have the ability to cross the placental barrier.[4]

Developmental toxicity of PFOA and PFOS has been demonstrated in rodents in particular regarding fetal growth, neonatal mortality and litter resorption in high doses. [5, 6] Epidemiologic investigations have explored associations between serum concentrations of PFOA and PFOS during pregnancy and adverse birth outcomes, including low birth weight, in exposed populations. The results are equivocal.[7] Four studies addressed miscarriage and stillbirths in Mid-Ohio Valley populations exposed to PFOA through contaminated drinking water and found no consistent link between the exposure and miscarriage or stillbirths.[8–11] A recent study including more than 300 miscarriages found no association between PFOA exposure and miscarriage but a tendency towards a positive association with PFOS exposure in the Mid-Ohio Valley population.[12] Exposure to other PFASs have not been considered. Thus, the present study examined exposure to PFOA, PFOS and the more recently introduced perfluorohexane sulfonic acid (PFHxS), perfluorononanoic acid (PFNA) and perfluorodecanoic acid (PFDA) which we have previously found in measurable levels in Danish populations in regard to miscarriage in a case-control study conducted within a population based cohort of Danish pregnant women.

## Materials and Methods

**Fig 1:**
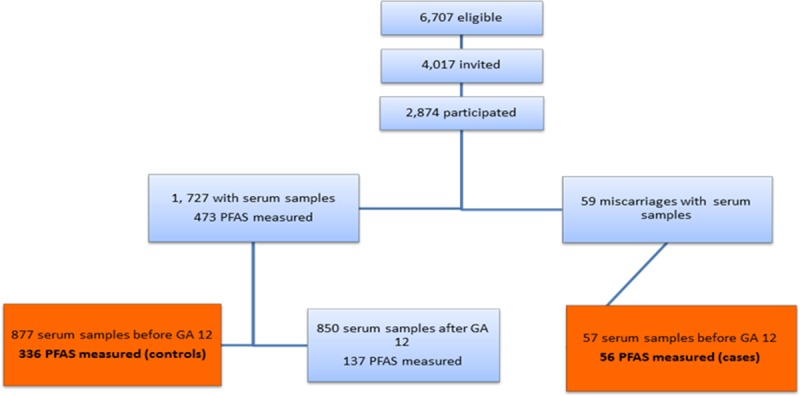
Recruitment and sampling strategy in the Odense Child Cohort.

The study was based on data from the Odense Child Cohort. [13] Briefly, newly pregnant women residing in the Municipality of Odense, Denmark between January 1, 2010 and December 31, 2012 were recruited at gestational age (GA) 8–16 weeks. Serum samples were collected at recruitment and stored at -80°C in freezers at the Odense Patient data Explorative Network (OPEN). Of the eligible population of 6,707 pregnant women, 4,017 who were informed declined participation while 2,874 (42.9%) enrolled in the cohort (Fig 1). Participants were better educated and more often of Danish origin than non-participants (manuscript in preparation).

All participants were enrolled before miscarriage occurred. Last menstrual period (LMP) was used to calculate the GA (in weeks) in all participants. Miscarriages were diagnosed at the hospital and the GA at the time of miscarriage was defined as the date of diagnosis by transvaginal ultrasound. A total of 88 miscarriages occurred before GA 22 weeks, and of which 59 had stored samples, we decided to include only those who donated a serum sample before GA 12, which was 57 miscarriages of which 56 had PFAS measured (Fig 1, cases). These were compared to delivering women of whom 1,727 donating a serum sample. As part of a biomonitoring project, [14] serum PFAS concentrations were measured in a random subset of 473 women of these. A total of 877 delivering women donated a serum sample before GA 12 weeks and of these 336 had PFAS measured in the biomonitoring project (controls) (Fig 1). In addition, we were able to match 51 of the cases to four controls on parity and days of gestation of serum sampling (plus or minus 2 days). Five cases could not be matched on days of gestation of serum sampling, as these occurred very early in pregnancy.

The LMP date, the pre-gestational body mass index (BMI), age at LMP, smoking and parity (number of previous births) were extracted from the patient chart at the first antenatal visit. Smoking was defined as smoking during early pregnancy regardless of quantity. The study complied with the Helsinki declaration and was approved by The health research ethics committee system in Denmark and the Danish Protection Agency (j.no. 2008-58-0035). The participants were informed in writing and verbally about the study. Written consent was obtained from all. The ethical committee approved this consent procedure.

### PFAS analysis

The blood samples were collected in BD Vacutainer serum collection tube by use of a BD Vacutainer Safety-Lok Blood Collection Set (Becton, Dickinson and Company, Franklin Lakes, NJ) or a Terumo Winged Blood Sampling Set with Holder and Protector (0.8 x 19 mm x 180 mm) (Terumo Europe, Leuven, Belgium) inserted into a cubital vein as recommended for blood sampling, and all sample preparation and storage, also of analytical standards, were uniform, as appropriate for PFAS analysis [15]. These have not been found to contain any of the quantified PFAS compounds or other persistent organic pollutants. [16] The blood collection sets consists of two steel cannulas, a short silicone tube plus some polypropylene connection fittings. Neither of these sets has been found to contain any of the quantified PFAS. The samples were centrifuged to serum, which was pipetted into polypropylene cryo tubes and immediately frozen to -80°C until analysis. As is standard in this field, we specifically avoided using sampling or storage tubes containing fluoropolymers (such as Teflon-coated materials) that could be a source of PFAS contamination [17]. Adhesion of long-chain PFASs has been reported only for aqueous solutions stored in glass and polypropylene containers [17], but our study does not include these PFASs. Although sample contamination or adsorption is always a possibility, our repeated analyses of serum samples over time as well as analyses of duplicate samples have shown excellent precision and have never revealed any indication of variance attributable to sampling and storage. While the samples were stored at -80°C for up to three years until analysis, the persistence of these compounds would suggest that any degradation would be negligible. We also note that our sampling, storage, and analytical procedures are similar to those that are considered best practice [15, 18].

The PFAS analysis was performed by on-line solid phase extraction followed by liquid chromatography and triple quadropole mass spectrometry (LC-MS/MS).[18] Laboratory reagent blanks, sample matrix blanks (horse serum) and fortified sample matrix blanks (horse serum) were included in all analytical runs in order to control for any contamination during the extraction process, as well as the precision of the method. Quality control and assessment samples from human serum stored at identical temperatures and in identical cryo tubes in a period covering the storage period of the analysed samples were also included in each analytical series. The following PFAS were quantified: PFOA, PFOS, PFHxS, PFNA, PFDA. The Limit of Quantification was 0.03 ng/mL for all substances, and values below this limit were replaced with half of the limit (i.e., 0.015 ng/mL). The analysis was performed at Dep. of Environmental Medicine, University of Southern Denmark, Denmark as described by Haug et al. (2009), with minor modifications. The analyses were performed in September 2011 and September 2013. For PFOA, PFOS, PFHxS, PFNA and PFDA, the within-batch coefficients of variation (CV) were <3% and the between-batch CV were <5.2%. For the metabolites, FOSA, MeFOSAA and EtFOSAA, the within-batch CV were <4.1%, whereas the between-batch CV were <6.8%. The accuracy and reliability of the data are continuously ensured by including quality control samples [e.g. from National Institute of Standards and Technology (NIST)] in each analytical batch of samples, calibration standards, along with reagent and serum blanks. Excellent results were obtained in the regular comparisons scheme organized by the German Society of Occupational Medicine (G-EQUAS). Inspection of the data from the quality assurance samples from NIST-1958 across a period of 3 months in which the reported samples also were analyzed showed the following average bias (measured concentration-target NIST concentration/target NIST concentration) x 100%) for the represented compounds: PFOS: +3%, PFOA: -7%, PFNA: -3% and PFHxS: +3%

### Statistical analysis

Serum-PFAS concentrations among women with and without miscarriage were first compared by the Mann Whitney test. Then multiple logistic regression was undertaken to calculate the odds ratio (OR) for miscarriage in regard to each of the PFAS as exposure variable (in tertile groups by exposure or transformed by the use of the natural logarithm due to the skewed distribution) while adjusting for age, BMI, parity, and GA at serum sampling as covariates as these were associated to PFAS exposure or independent risk factors for miscarriage. In addition, conditional logistic regression in the matched dataset was performed. We also included all PFAS in the same model and repeated the analyses using stratified data according to parity. ORs are presented with 95% confidence intervals (95% CI).

## Results

All PFAS were detected in almost all serum samples, with a median (5–95 percentiles) for PFHxS of 0.29 ng/mL (0.02–7.28); for PFOA, 1.58 ng/mL (0.31–9.71); for PFOS, 8.10 ng/mL (1.25–26.12), for PFNA, 0.72 ng/mL (0.18–4.40) and for PFDA, 0.27 ng/mL (0.07–1.75). Only PFHxS showed serum concentrations below level of detection (LOD), in 4% of the samples, while all other PASs were detected in all samples; all PFAS were highly correlated (p<0.01 for all). A total of 473 delivering women had PFAS measured of which 336 donated serum samples before GA week 12 (Fig 1). PFAS levels did not differ between the 336 included women (controls) and the 137 women with serum samples donated apart from PFHxS which was higher in the women not included (data not shown).

Median concentrations of PFNA and PFDA were higher among women with miscarriage compared to women without miscarriage (Table 1). First pregnancies and women with BMI within the normal range had higher serum concentrations of PFAS, whereas no association was found with smoking, age or gestational age at serum sampling apart from PFOA concentration which was higher in older and smoking women.

**Table 1:** Association between lifestyle and reproductive factors and serum concentrations of PFASs (median (M) and 5^th^ and 95^th^ percentiles(5–95)) among 392 pregnant women from Odense, Denmark 2010–2012.

Variable	N	PFHxS ng/mL		PFOS ng/mL		PFOA ng/mL		PFNA ng/mL		PFDA ng/mL	
		M	5–95	M	5–95	M	5–95	M	5–95	M	5–95
Miscarriage											
No	336	0.28	0.05–0.66	7.85	3.64–15.79	1.59	0.62–4.51	0.68	0.31–1.53*	0.26	0.15–0.56*
Yes	56	0.34	0.14–0.63	8.77	3.89–15.22	1.46	0.51–3.15	1.16	0.63–2.46	0.33	0.17–0.66
Age (years)											
<30	201	0.29	0.06–0.56	8.24	3.68–16.80	1.79*	0.71–4.46	0.76	0.30–1.98	0.27	0.13–0.61
30–34	126	0.28	0.05–0.68	7.06	4.12–14.66	1.51	0.52–3.68	0.65	0.32–1.55	0.26	0.17–0.53
≥35	65	0.32	0.06–0.90	7.48	3.52–14.34	1.26	0.60–4.65	0.78	0.33–1.98	0.27	0.16–0.76
Smoking											
No	378	0.29	0.06–0.63	8.04	4.64–15.71	1.58*	0.57–4.27	0.73	0.31–0.61	0.27	0.16–0.59
Yes	14	0.27	0.04-	8.65	4.18-	2.03	1.23-	0.61	0.35-	0.22	0.15-
BMI (kg/m^2^)											
<20	41	0.26	0.02–0.72*	8.99	2.96–14.93	1.59	0.49–3.54	0.70	0.29–1.51*	0.26	0.16–0.57
20–24.9	187	0.32	0.06–0.68	8.39	3.91–17.69	1.74	0.61–4.66	0.78	0.35–1.74	0.28	0.16–0.61
≥25	164	0.28	0.07–0.56	7.42	3.59–14.41	1.52	0.56–3.88	0.64	0.29–1.94	0.25	0.14–0.52
Parity											
1st pregnancy	219	0.36	0.08–0.72*	9.05*	4.21–16.81	2.01*	0.83–4.63	0.79	0.37–1.88*	0.28	0.16–0.62*
2nd or more	173	0.22	0.03–0.51	6.64	3.45–14.87	1.18	0.52–3.11	0.64	0.29–1.42	0.25	0.14–0.48
Gestational age at serum sampling (weeks)											
<10	195	0.28	0.05–0.59	7.94	3.64–15.16	1.57	0.58–4.20	0.73	0.29–1.65	0.27	0.15–0.58
≥10	197	0.29	0.07–0.66	8.13	3.65–17.53	1.61	0.57–4.50	0.72	0.35–1.85	0.27	0.16–0.60

* Mann Whitney test of differences between distributions p<0.05

In an unadjusted logistic regression, a significant trend was found for miscarriage OR in regard to PFNA and PFDA tertiles. Covariate adjustment had little impact on these associations (Table 2). After adjustment for age, BMI, parity and gestational age at serum sampling, women with miscarriage had ORs of 16.5 (95% CI 7.4–36.6–36.5) and 2.67 (1.31–5.44), respectively, of being in the highest tertile of PFNA and PFDA exposure, respectively. In conditional logistic regression in the matched dataset the OR were respectively 37.9 (9.9–145.2) and 3.71 (1.60–8.60). The estimates had wide CIs due to the small number of miscarriages that required the use of natural logarithmic transformed continuous estimates. After inclusion of all five PFAS in the model, PFNA continued to show a highly significant association with miscarriage. These associations were similar among nulli- and multiparious women. No significant associations were seen between PFOA and PFOS and miscarriage.

**Table 2:** Unadjusted and adjusted odds ratio (OR) and 95% confidence interval (95% CI) for miscarriage according to PFAS exposure as a continuous variable and in tertiles among 392 pregnant women from Odense, Denmark 2010–2012.

PFAS exposure (ng/mL)	Case-cohort design					Matched case-control design			
	N Miscarriage/birth	Unadjusted OR for miscarriage		Adjusted^1)^ OR for miscarriage		Unadjusted OR for miscarriage		Adjusted^1)^ OR for miscarriage	
		OR	95% CI	OR	95% CI	OR	95% CI	OR	95% CI
**PFHxS**									
1^st^ tertile	19/142	Reference		Reference		Reference		Reference	
2^nd^ tertile	18/95	1.43	0.72–2.85	1.46	0.72–3.00	1.46	0.68–3.16	1.30	0.58–2.89
3^rd^ tertile	19/99	1.42	0.71–2.84	1.46	0.68–3.13	1.73	0.80–3.78	1.53	0.67–3.51
Continuous^2)^	56/336	1.47	0.98–2.21	1.53	0.99–2.38				
**PFOS**									
1^th^ tertile	19/128	Reference		Reference		Reference		Reference	
2^th^ tertile	18/94	1.12	0.57–2.23	1.15	0.57–2.35	1.12	0.52–2.40	1.03	0.47–2.29
3^th^ tertile	19/114	1.29	0.64–2.59	1.33	0.64–2.78	1.25	0.57–2.71	1.06	0.46–2.41
Continuous^2)^	56/336	1.12	0.59–2.12	1.16	0.59–1.29				
**PFOA**									
1^st^ tertile	19/98	Reference		Reference		Reference		Reference	
2^nd^ tertile	18/122	1.18	0.59–2.36	1.23	0.60–2.51	1.15	0.53–2.51	1.21	0.53–2.73
3^rd^ tertile	19/116	0.90	0.45–1.80	0.93	0.42–2.03	0.69	0.29–1.62	0.68	0.28–1.67
Continuous^2)^	56/336	0.67	0.41–1.10	0.64	0.36–1.18				
**PFNA**									
1^th^ tertile	18/274	Reference		Reference		Reference		Reference	
2^th^ tertile	19/28	8.51	4.07–17.77	10.88	4.76–24.84	17.55	5.73–53.75	18.97	5.49–65.58
3^th^ tertile	19/34	10.33	4.87–21.93	16.17	6.88–38.03	32.06	9.31–110.36	37.93	9.90–145.2
Continuous^2)^	56/336	11.82	5.78–24.19	16.46	7.39–36.62				
**PFDA**									
1^th^ tertile	19/180	Reference		Reference		Reference		Reference	
2^th^ tertile	19/70	1.98	0.99–3.97	1.86	0.91–3.83	1.89	0.86–4.17	1.85	0.77–4.46
3^th^ tertile	18/86	2.57	1.29–5.14	2.67	1.31–5.44	3.08	1.45–6.57	3.71	1.60–8.60
Continuous^2)^	56/336	2.29	1.20–4.39	2.30	1.18–4.47				

In addition, conditional logistic regression on 1:4 matched data (on parity and gestational week at inclusion) with 51 cases of miscarriage and 204 controls.

^1.^ Adjusted for age, BMI, parity and gestational age at serum sampling.

^2.^ Transformed by the use of the natural logarithm. *p<0.05

## Discussion

In this case-control study we found strongly significant associations between serum concentrations of PFAS (PFDA and especially, PFNA) and miscarriage and almost significant association with PFHxS exposure. PFOA and PFOS occurred in lower concentrations than in previous studies,[8–12] as a likely consequence of their decreased production, which may explain why we did not find association between PFOA and PFOS exposure and miscarriage. However, the more recently introduced PFAS were found in higher concentrations than previously reported. Despite the low number of miscarriages included (N = 56), significant trends and high ORs were found for serum PFAS concentrations in the third tertile. As these findings showed no impact of known risk factors, even after matching on parity and gestational age of serum sampling, these results may suggest causal associations although residual confounding from lifestyle and behavioral factors (socioeconomic status, diet etc.) may be a possible explanation. Still, due to the small number of miscarriages included, the confidence intervals were wide.

Reproductive toxicity of PFOA and PFOS has been demonstrated in animal studies with increased neonatal mortality and decreased fetal growth and reduced litter size in high concentrations,[5, 6] but the adverse effects of the more recently introduced PFAS is largely unknown. In a population in the Mid-Ohio Valley with high PFOA exposure, no consistent link to miscarriage and stillbirths was found [8–12] although a suggestive association with high PFOS exposure was found especially in first pregnancies after PFAS measurement, which is in accordance with our findings at much lower levels of exposure. The Ohio Valley studies [8–12] were much larger than ours and adjusted for more potential confounders but relied on self-reporting of miscarriage. In addition, pregnancies and miscarriages occurred either on average 1.8 years after the measurement of PFAS exposure[12] or PFAS levels at the time of pregnancy was estimated as pregnancies occurred up to 15 years before the PFAS measurements. [8–11] While some studies also considered PFOS, we have been unable to find any previous studies on adverse reproductive effects of PFHxS, PFNA and PFDA exposure.

Both unrealized miscarriages and those that occurred before contact with the health care system were not included in the present study. We included only women donating a serum sample before GA week 12 as most miscarriages occur in first trimester and therefore women included after GA week 12 have survived the first trimester risk and have a lower risk of subsequent miscarriage. However, we compared serum levels of PFAS among the women delivering a serum sample before and after GA week 12 and found no differences apart from PFHxS. Our study was prospective and relied on a baseline serum sample and subsequent documentation of miscarriage but differences in pharmacokinetics between pregnancies ending in life births and miscarriages may affect our findings. [19] Of those eligible 42.9% participated, and 32.1% of the participants supplied an early pregnancy serum sample.

Parity was strongly associated with PFAS exposure and may also be a function of reproductive success, which makes it difficult to adjust for as cases may have experienced more miscarriages than controls. We therefore performed analyses by matching on parity and gestational age of serum sampling, which did not change the findings. In addition, the findings were similar when stratifying by parity. While the participants may have had a larger number of previous miscarriages, it is unlikely that they were associated with their PFAS levels. We cannot exclude residual confounding or confounding by certain lifestyle or behavior associated with PFAS exposure, as seafood intake and food packaging materials [2, 3] are exposure sources and therefore dietary habits, simultaneous exposure to other chemicals (e.g. methylmercury, other endocrine distupting chemicals) as well as socioeconomic status may be confounders, which may be a likely explanation to our findings.

In conclusion, our study suggests an association between miscarriage and serum concentrations of PFAS, especially the more recently used PFDA, PFHxS and especially PFNA, which were found in higher concentrations than in previous studies. No consistent pattern was found for PFOA or PFOS, which may be due to lower exposure than in previous studies. These findings are of potential public health importance, as all pregnant women were exposed to PFAS compounds. However, our study was based on 56 miscarriages and residual confounding is possible and findings need confirmation.
