# Correction: Association between Perfluorinated Compound Exposure and Miscarriage in Danish Pregnant Women

**DOI:** 10.1371/journal.pone.0149366

**Published:** 2016-02-09

**Authors:** Tina Kold Jensen, Louise Bjørkholt Andersen, Henriette Boye Kyhl, Flemming Nielsen, Henrik Thybo Christesen, Philippe Grandjean

In Table 2, there are errors in the “N Miscarriage/birth” column. Please see the corrected Table 2 here.

**Table 2:** Unadjusted and adjusted odds ratio (OR) and 95% confidence interval (95% CI) for miscarriage according to PFAS exposure as a continuous variable and in tertiles among 392 pregnant women from Odense, Denmark 2010–2012.

PFAS exposure (ng/mL)	Case-cohort design					Matched case-control design			
	N Miscarriage/birth	Unadjusted OR for miscarriage		Adjusted^1^^)^ OR for miscarriage		Unadjusted OR for miscarriage		Adjusted^1^^)^ OR for miscarriage	
		OR	95% CI	OR	95% CI	OR	95% CI	OR	95% CI
PFHxS									
1^st^ tertile	19/142	Reference		Reference		Reference		Reference	
2^nd^ tertile	19/99	1.43	0.72–2.85	1.46	0.72–3.00	1.46	0.68–3.16	1.30	0.58–2.89
3^rd^ tertile	18/95	1.42	0.71–2.84	1.46	0.68–3.13	1.73	0.80–3.78	1.53	0.67–3.51
Continuous^2^^)^	56/336	1.47	0.98–2.21	1.53	0.99–2.38				
PFOS									
1^th^ tertile	19/128	Reference		Reference		Reference		Reference	
2^th^ tertile	19/114	1.12	0.57–2.23	1.15	0.57–2.35	1.12	0.52–2.40	1.03	0.47–2.29
3^th^ tertile	18/94	1.29	0.64–2.59	1.33	0.64–2.78	1.25	0.57–2.71	1.06	0.46–2.41
Continuous^2^^)^	56/336	1.12	0.59–2.12	1.16	0.59–1.29				
PFOA									
1^st^ tertile	19/116	Reference		Reference		Reference		Reference	
2^nd^ tertile	19/98	1.18	0.59–2.36	1.23	0.60–2.51	1.15	0.53–2.51	1.21	0.53–2.73
3^rd^ tertile	18/122	0.90	0.45–1.80	0.93	0.42–2.03	0.69	0.29–1.62	0.68	0.28–1.67
Continuous^2^^)^	56/336	0.67	0.41–1.10	0.64	0.36–1.18				
PFNA									
1^th^ tertile	18/274	Reference		Reference		Reference		Reference	
2^th^ tertile	19/34	8.51	4.07–17.77	10.88	4.76–24.84	17.55	5.73–53.75	18.97	5.49–65.58
3^th^ tertile	19/28	10.33	4.87–21.93	16.17	6.88–38.03	32.06	9.31–110.36	37.93	9.90–145.2
Continuous^2^^)^	56/336	11.82	5.78–24.19	16.46	7.39–36.62				
PFDA									
1^th^ tertile	19/180	Reference		Reference		Reference		Reference	
2^th^ tertile	18/86	1.98	0.99–3.97	1.86	0.91–3.83	1.89	0.86–4.17	1.85	0.77–4.46
3^th^ tertile	19/70	2.57	1.29–5.14	2.67	1.31–5.44	3.08	1.45–6.57	3.71	1.60–8.60
Continuous^2^^)^	56/336	2.29	1.20–4.39	2.30	1.18–4.47				

In addition, conditional logistic regression on 1:4 matched data (on parity and gestational week at inclusion) with 51 cases of miscarriage and 204 controls.

1 Adjusted for age, BMI, parity and gestational age at serum sampling.

2 Transformed by the use of the natural logarithm. *p<0.05
